# The Sowers of Seeds: A Qualitative Analysis of the Role of Palliative Care Educators in Facilitating Goals-of-Care Conversations and Palliative Care Referrals

**DOI:** 10.1177/10499091241267917

**Published:** 2024-08-28

**Authors:** Seth N. Zupanc, Lisa M. Quintiliani, Amy M. LeClair, Michael K. Paasche-Orlow, Angelo Volandes, Akhila Penumarthy, Lori Henault, Jennifer E. Itty, Aretha D. Davis, Joshua R. Lakin

**Affiliations:** 1 Department of Psychosocial Oncology and Palliative Care, 1855 Dana-Farber Cancer Institute, Boston, MA, USA; 2 School of Medicine, 8785 University of California San Francisco, San Francisco, CA, USA; 3 Division of General Internal Medicine, Department of Medicine, Tufts University School of Medicine, Tufts Medical Center, Boston, MA, USA; 4 1811 Harvard Medical School, Boston, MA, USA; 5 Section of General Internal Medicine, 2348 Massachusetts General Hospital, Boston, MA, USA; 6 ACP Decisions, Newton, MA, USA; 7 Section of General Internal Medicine, 1836 Boston Medical Center, Boston, MA, USA; 8 Institute of Health System Science, 88982 Northwell Health Feinstein Institutes for Medical Research, Manhasset, NY, USA; 9 Division of Palliative Medicine, 1861 Brigham and Women’s Hospital, Boston, MA, USA

**Keywords:** palliative care, advance care planning, implementation science, qualitative interviews, communication, palliative care educator

## Abstract

**Background:**

Optimal care for seriously ill and older patients often involves advance care planning (ACP), goals-of-care (GOC) conversations, and specialty palliative care consultation, three sometimes overlapping, yet distinct practices. Insufficient staffing and investment in these areas have limited their availability.

**Objectives:**

We explored the facilitators and barriers to successful implementation of the VIDEO-PCE trial. The intervention aimed to increase patient engagement in ACP, GOC, and by establishing Palliative Care Educators, a new clinical role integrated into existing hospital wards.

**Design:**

This qualitative interview study employed a semi-structured interview guide tailored to the interviewee’s clinical role. The interviews elicited perceptions of the facilitators and barriers to integration of palliative care educators (PCEs) into existing workflows. We developed deductive codes a priori and inductive codes as we coded interview transcripts.

**Setting/Subjects:**

Medical/surgical floor clinical colleagues, palliative care team members, and PCEs from both participating sites were interviewed.

**Results:**

Twenty-four individuals were interviewed (12 clinical staff of medical and surgical wards, seven palliative care team members, and five PCEs). Four themes were identified: (1) The work completed by the PCEs provided a foundation for future palliative care involvement; (2) Constituting the new role in practice required revision and creativity; (3) Communication was important to providing continuity of care; and (4) Establishing trust catalyzed the acceptance of the role.

**Conclusion:**

The creation and implementation of a new role within existing clinical workflows posed some challenges but were felt to relieve staff from some work burden and allow more patients to engage in ACP and GOC conversations.

**Trial Registration:**

ClinicalTrials.gov Identifier: NCT04857060.

## Background

Although sometimes confounded as synonyms, advance care planning (ACP), goals-of-care (GOC) conversations, and specialty palliative care consultations represent unique, sometimes overlapping, spheres of need for patients, especially those with a serious illness.^1-3^ Advance care planning is defined as the dynamic, ongoing communication and planning process to prepare patients and their caregivers to consider their options for future medical care while grounded in what matters most to the patient.^4-6^ Goals-of-care conversations represent discussions that elicit a person’s values and priorities to guide medical decision making.^7^ Palliative care is a medical specialty focused on alleviating symptoms and stress associated with serious illness, improving quality of life, and assisting patients and caregivers in medical decision making.^8^

While programs have been promulgated to provide ACP discussions, GOC conversations, and interprofessional palliative care consultations, these efforts have not kept pace with the increasing demand for each intervention.^9-18^ Due to barriers such as insufficient financial investment, staffing shortages, and overburdened clinicians, the benefits of ACP, GOC conversations, and specialty palliative care go unrecognized for many patients, and disproportionally so for patients of a minoritized identity.^19-22^

The Video Images about Decisions to Improve Ethical Outcomes with Palliative Care Educators (VIDEO-PCE) trial sought to address barriers to conducting hospital-based ACP and GOC and delivering specialty palliative care through creating a new role for the hospital: dedicated palliative care educators (PCEs).^23^ Palliative care educators were nurses and social workers trained to engage patients and their surrogates in ACP and GOC conversations, collaborate with clinical teams to communicate elicited information, and facilitate palliative care consultations as needed. Their role was designed as a proactive hospital service for older patients and all patients with dementia, not contingent on a referral request to be ordered by the treating service. The PCEs were connected into palliative care teams, trained in serious illness communication, and provided a suite of video decision aids designed to educate patients on ACP, GOC, and palliative care topics.^23-25^ Previously, in the primary analysis of trial data, the intervention significantly increased documentation of conversations about goals, limitations of life-sustaining treatment, palliative care, hospice, and time-limited trails, especially for African American, Hispanic, and non-English speaking patients, as well as for people living with Alzheimer disease and related dementias.^26^

In the current qualitative study, we explore the facilitators and barriers to successful integration of the PCEs into existing clinical workflows. Lessons learned from the VIDEO-PCE trial can guide the creation and implementation of future interventions to improve ACP, GOC, and palliative care, as well as interventions in other domains that utilize a proactive model akin to PCEs to improve hospital care.

## Methods

### Study Design and Context

The VIDEO-PCE trial was a pragmatic stepped-wedge cluster randomized trial conducted at Boston Medical Center (Boston, MA) and North Shore University Hospital (Manhasset, NY), between July 2021 and October 2022.^23^ The trial’s intervention involved the integration of PCEs—nurses and social workers who received specialized training in facilitating ACP, eliciting GOC, and collaborating with specialty palliative care—into study inpatient units (hospital wards) at each hospital. We have previously published a full description of the primary outcomes and study design—including detailed descriptions of the training, support, and tools used by the PCEs.^23,26^

For this qualitative study aimed at describing and characterizing facilitators and barriers to PCE implementation, we conducted semi-structured interviews with two groups of individuals (1) palliative care team members, and (2) clinical staff of medical and surgical wards (ie, nurses, social workers, physicians; henceforth referred to as ‘medical/surgical floor colleagues’) from study units. Because of their shared experiences crafting workflows, we conducted small group discussions with the PCEs – one at each of the two hospitals – with the goal of eliciting shared experiences through the discussion; other clinical staff members were interviewed individually.

### Participants

All PCEs, members of specialty palliative care teams, and clinical staff working on study units were eligible for participation. Research managers sent recruitment emails providing information about the study and asking them to contact a member of the study team (JRL) to enroll. In total, 62 Medical/Surgical Floor Colleagues and 12 Palliative Care Team Members were invited to participate in an interview. Seven PCEs—three from one site and four from the second site—were invited to participate in site-specific small group discussions. We aimed to recruit a sample of 24 participants comprised of five to seven PCEs, two to five individuals from site palliative care teams, and twelve medical/surgical floor colleagues.

### Data Collection and Analysis

We developed a semi-structured interview guide based on ACP, GOC, and palliative care literature and our team’s experience crafting the VIDEO-PCE trial.^23,27-30^ The interview guides were tailored to the participant’s role (i.e., PCE, palliative care team member, medical/surgical floor colleague) to elicit facilitators and barriers to successful integration of the PCEs into existing hospital wards (Appendix). We conducted all interviews via Zoom and, with the participant’s consent, recorded both audio and video for later verbatim transcription. Study staff (JRL and SNZ) with expertise in palliative care conducted all interviews. Participants were not compensated for their participation. Audio transcripts were professionally transcribed and reviewed for accuracy by study team members and uploaded into NVivo (release 1.7.1), Our codebook was comprised of both deductive and inductive codes. Deductive codes were developed a priori based on the semi-structured interview guide and input from clinical experts. Inductive codes were iteratively developed as interview transcripts were coded. Initially, three transcripts from individual interviews were double coded (JRL and SNZ) to ensure the codebook was being applied consistently and to resolve definitional discrepancies. The remaining 16 individual interview transcripts with palliative care team members and medical/surgical floor colleagues were independently coded (8, JRL; 8, SNZ) with the two coders meeting regularly to discuss coding process, application, and findings. Any discrepancies in coding were resolved via comparison and consensus.^31^ Both small group discussion transcripts were double coded. After all transcripts were coded, two investigators (JRL and SNZ) conducted a thematic analysis by independently reviewing the exported quotations and results for each code.^31^ Throughout this process, JRL and SNZ met regularly with two experts in qualitative methods (LQ and AL) regarding emergent themes and findings to conduct peer debriefing to enhance analysis credibility.^32^ During these meetings, patterns among codes were discussed and summarized into themes, reported below. The Institutional Review Board at Dana-Farber Cancer Institute approved study procedures (IRB Number: 22-459).

## Results

Twenty-four people were interviewed: five PCEs (71.4%), seven palliative care team members (58.3%), and 12 (19.4%) medical/surgical floor colleagues (Table 1). Two small group discussions (Group 1, n = 2; Group 2, n = 3) with PCEs were completed and 19 individual interviews were conducted with palliative care team members and medical/surgical floor colleagues. On average, individual interviews lasted for 23 minutes and ranged between 14 and 33 minutes. The small group discussions were 53 and 58 minutes in length. Our analysis reached the point of thematic saturation and led to the creation of four themes.

**Table 1.** Characteristics of Interview Participants.^a^

	Site 1 (n = 14)	Site 2 (n = 10)	Total
Current title, n (%)			
Palliative care and/or geriatrics physician	2 (14%)	2 (20%)	4 (17%)
Other physician	1 (7%)	3 (30%)	4 (17%)
Clinical social worker	0 (0%)	1 (10%)	1 (4%)
Palliative care nurse/Nurse practitioner	0 (0%)	2 (20%)	2 (8%)
Other nurse/Nurse practitioner	5 (36%)	0 (0%)	5 (21%)
Palliative care educator	3 (21%)	2 (20%)	5 (21%)
Managers/Program managers	3 (21%)	0 (0%)	3 (13%)
Professional training, n (%)			
Physician	3 (21%)	4 (40%)	7 (29%)
Social worker	2 (14%)	4 (40%)	6 (25%)
Nurse/Nurse practitioner	8 (57%)	2 (20%)	10 (42%)
Administrative	1 (7%)	0 (0%)	1 (4%)
Years since finishing professional training mean (sample range)	14.4 (2-35)	13.0 (2-25)	13.8 (2-35)
Years at facility, mean (sample range)	8.9 (0.8-25)	6.9 (1-20)	8.1 (0.8-25)
Years in current role, mean (sample range)	4.3 (0.5-17)	3.0 (0.4-9)	3.8 (0.4-17)
Race, n (%)			
African American	3 (21%)	0 (0%)	3 (13%)
Asian	3 (21%)	2 (20%)	5 (21%)
White/Caucasian	7 (50%)	7 (70%)	14 (58%)
Other/Reported mixed	1 (7%)	1 (10%)	2 (8%)
Ethnicity, n (%)			
European/Caucasian/American/White	7 (50%)	4 (40%)	11 (46%)
Asian/Asian American	3 (21%)	4 (40%)	7 (29%)
Caribbean	1 (7%)	0 (0%)	1 (4%)
African American/Black	3 (21%)	0 (0%)	3 (13%)
Hispanic/Latino	0 (0%)	1 (10%)	1 (4%)
Not specified	0 (0%)	1 (10%)	1 (4%)
Primary language of English, n (%)	14 (100%)	10 (100%)	24 (100%)
Additional languages, n (%)			
Speaks one or more additional languages	6 (43%)	3 (30%)	9 (38%)
Speak no additional languages	8 (57%)	7 (70%)	15 (63%)
Gender, %			
Female	12 (86%)	8 (80%)	20 (83%)
Male	2 (14%)	2 (20%)	4 (17%)

^a^All data in this table was self-reported with open ended questions and summarized to provide anonymity.

### Thematic Results

The VIDEO-PCE study intervention employed PCEs to engage patients and their surrogates proactively in GOC communication.^26^ Palliative care educators were responsible for preparing patients and their surrogates for such conversations. While PCEs were given some guidance about how to fulfill their role (i.e., trainings, video decision support tools, electronic medical record note templates for documentation), they were also given flexibility to manage their daily activities to fit the workflow of the health systems in which they were operating.

### Theme 1: Providing a Foundation for Better Palliative Care

Interview respondents reported appreciating the multiple ways PCEs created a foundation for future specialty palliative care involvement and were described by a palliative care manager as the *“sower of seeds.”* Respondents described the tasks completed by the PCEs such as *“*[gathering] *baseline illness understanding”* (Participant 2, Palliative Care Team Member), *“identify(ing) specific palliative care needs,*” (Participant 4, Palliative Care Team Member) and *“educating the* [medical/surgical floor] *teams and empowering the teams to put in the* [palliative care] *consult.”* (Participant 12, Palliative Care Team Member) one medical/surgical floor team member noted gratitude for this work as it mitigated downstream stress by preventing their team from having to *“make those last-minute calls to the palliative care team.”* (Participant 14, Medical/Surgical Floor Colleague).

By educating and engaging patients early in GOC conversations, the PCEs laid foundations from which other clinical team members could extend in subsequent conversations. One medical/surgical floor colleague described this perceived impact on patients, noting the PCEs helped patients *“feel less jumpy”* about someone from the palliative care team subsequently visiting them. (Participant 5, Medical/Surgical Floor Colleague) The PCEs were seen as facilitating more efficient palliative care work by alleviating some of the teams’ workload. For example, through their work doing patient engagement and subsequent documentation the PCEs accomplished tasks that *“*[could] *take hours for the provider”* (Participant 12, Palliative Care Team Member).

### Theme 2: Building and Revising the Program

While the tasks and activities of the PCEs facilitated future palliative care involvement, we observed a theme describing the complexity of creating this new role. While flexibility inherent to the PCE position was noted to be an important contributor to success, it may have also contributed to confusion about the role of the PCE. For example, palliative care teams members at the same site described notably different integration of the PCEs with the palliative care team – one stated they *“absolutely”* felt the PCEs were members of the palliative care team (Participant 20 Palliative Care Team Member) while another described not seeing PCEs as members of their team. Even the inclusion of ‘palliative care’ in the name ‘Palliative Care Educator’ seemed to confuse some, with one palliative care team member reporting PCE involvement with a patient was sometimes confused for specialty palliative care team involvement.

To preemptively address confusion about the trial, the PCEs and site research team members would meet with unit teams to introduce the role, the research, and answer questions prior to engaging patients on that unit. Though the PCEs noted this early engagement and education with colleagues *“needed to occur”* (PCE Small Group Discussion 1), some medical/surgical floor colleagues indicated they would have preferred more education to *“make sure that what* [the PCEs] *were doing was truly valuable work and being done in the right way”* (Participant 16, Palliative Care Team Member). Moreover, while they were distributed in floor orientations by the study team, some medical/surgical floor colleagues expressed interest in accessing the resources (ie, video decision aids and accompanying handouts) the PCEs utilized (Participants 17 and 25, Medical/Surgical Floor Colleagues).

Despite preemptive efforts to introduce and alleviate confusion around the PCE role, a lack of clarity about the purpose and the specific tasks of the PCEs was a commonly cited source of frustration for medical/surgical floor colleagues. The PCEs also noted some frustration when their role was not well understood by medical/surgical floor colleagues, with one PCE stating they felt “*…like I had to do my due diligence to touch base with teams before I went in to see a patient,”* but when they touched base with the clinical team, “*all providers would be like, that’s okay, you don’t need to do that, I will take care of that.”* As a result, that PCE explained, “*I felt frustrated by the fact that sometimes primary care teams did not see the value”* (PCE Small Group Discussion 1).

Additionally, PCEs and medical/surgical floor colleagues noted some practical barriers that could have also been addressed through better resources such as providing a physical office space in the hospital and reliable access to internet throughout the hospital units for showing study videos on iPads. This was despite the fact that internet access was not needed to show videos via the application. On the other hand, the PCEs pointed to scheduled coaching calls (monthly supportive meetings with two palliative care physicians from outside of the intervention hospital systems), the availability of study staff, and the guidance of clinical supervisors as sources of support as they learned to navigate their role (PCE Small Group Discussion 1).

The PCEs also navigated uncertainty in developing specific skills. For example, finding ways to engage patients effectively and efficiently in GOC conversations was a skill that took several of the PCEs some time to develop. Logistically, one PCE noted a challenge in *“figuring out the best time to see patients.”* (PCE Small Group Discussion 2) Another PCE spoke to having to find a way to shape their encounters so that important information was being elicited in a time-efficient manner (PCE Small Group Discussion 1). Despite some of the challenges described above, PCEs noted that there was success and joy to be found in the flexibility of the role.

### Theme 3: Ensuring Successful Communication and Completion of Clinical Tasks

Respondents described that establishing a new role required the revision of existing communication and information sharing patterns; however, the mechanisms for establishing successful new communication patterns were not always clear. To address barriers to communication and facilitate the workflow, the research team created a note template for PCE patient encounters, and some palliative care team members and medical/surgical floor colleagues reported seeing and utilizing the notes left by PCEs (Participant 12, Palliative Care Team Member; Participant 13, Medical/Surgical Floor Colleague). In contrasting comments about how PCEs communicated beyond notes, one medical/surgical floor colleague indicated the PCEs would *“check in”* to *“let me know what happened”* (Participant 1, Medical/Surgical Floor Colleague), while another indicated quicker, in-person debriefings would have been helpful (Participant 26, Medical/Surgical Floor Colleague). One PCE noted provider hand-offs could be used as a way of *“seeing where the patient is, what’s been done,* […and gathering] *information that would be beneficial for us [the PCEs]*.*”* (PCE Small Group Discussion 2). Additionally, several respondents pointed to numerous electronic communications platforms as modes for PCE follow-up.

Relatedly, many respondents described tension around confirming the completion of a clinical task. There was a persistent tension around the completion of MOLST/POLST forms, related to legal limits on what kinds of professionals are able to officially sign the forms. While some colleagues were grateful the PCEs helped to elicit the patient’s wishes *“prior to any unfortunate events”* (Participant 14, Medical/Surgical Floor Colleague), the process of codifying wishes after a PCE interaction into medical orders was often described a difficult one for the clinical staff. Given that PCEs often engaged patients in conversations around treatment intensity preferences, patients often expressed a desire to clarify and document their preferences via a MOLST/POLST form following the PCE intervention (Participant 16, Palliative Care Team Member). Some colleagues voiced a frustration they felt about needing to *“go in behind* [the PCE’s conversation] *and reiterate or repeat what they have said”* because the PCEs were unable to complete a MOLST form due to their clinical credentialing (Participant 11, Medical/Surgical Floor Colleague).

### Theme 4: Building Trust while Navigating Varied Patient and Provider Cultures

To fulfill their role, PCEs had to navigate the organizational culture on different hospital units, while striving to foster trust with patients and colleagues. In speaking about steps taken to build trust, a medical/surgical floor colleague described the importance of familiarity with the PCEs so *“*[it wasn’t] *just like random strangers coming through and having these conversations with our patients”* (Participant 5, Medical/Surgical Floor Colleague). Likewise, the PCEs noted *“once we established those relationships, that’s when we really thrived on the floors”* (PCE Small Group Discussion 2). However, doing so involved PCEs navigating varying unique cultures. Some units *“received [them] with open arms,”* while the PCEs reported other units in which they *“did not have a lot of foot traffic”* (PCE Small Group Discussion 2).

## Discussion

In this qualitative interview study exploring facilitators and barriers to successful integration of PCEs during the VIDEO-PCE trial, we identified several themes describing the implementation and impact of this new role. First, we observed work done by PCEs laid a foundation for patients, caregivers, medical/surgical floor colleagues, and palliative care teams to build upon in subsequent ACP discussions, GOC conversations, and palliative care consultations. This unfolded in expressions of gratitude for saved time, avoided stress, and expedited impactful work. Second, we observed tensions about the creation of this new role and how it was situated between medical/surgical floor colleagues and palliative care teams. As a new role, care was needed to confirm that communication was successful and that tasks were completed. Lastly, PCEs learned to navigate the complex cultural landscapes of individual hospital units where they worked.

Palliative care staff shortages and the large volume of unmet palliative care needs have been used as arguments for training clinicians without specialty palliative care training to facilitate ACP and GOC conversations.^33,34^ However, staffing, burnout, time constraints, and other barriers to completing ACP and GOC conversations exist for these clinicians as well.^35-37^ As such, the VIDEO-PCE trial aimed to introduce a new integrated proactive role to facilitate ACP and GOC conversations for hospitalized older adults and efficient use of specialty palliative care. We found that interview participants reported the role was well-received and achieved its goal of creating efficiency, interpersonal bonds, and improving palliative care work. At the same time, we observed the creation of this new role generated some friction that required attention.

Many of the facilitators and barriers we uncovered are consistent with those outlined in a 2018 systematic review of hospital-based interventions.^38^ Like in that review, our interviews revealed that creating a supportive context (i.e., proper physical and cultural space), ensuring that staff understood the importance of the intervention and were not additionally burdened by it, and adapting existing systems to change were crucial components to facilitating success. Uniquely, the VIDEO-PCE intervention integrated a new position into existing workflows rather than adding new workflows into existing infrastructure. An advantage of this approach is that it did not directly burden staff with additional tasks. However, a disadvantage of this approach is the effort necessary to embed a new role into hospital culture and practices.

Our study has several limitations. First, it was conducted at two research sites, both academic medical centers with palliative care and an openness to an intervention that created a novel palliative care-adjacent role, which may limit generalizability. Second, both centers were in large metropolitan areas in the Northeast United States, again limiting generalizability. Last, while we invited participants broadly, our sample may reflect the views of people who were more engaged with the study or otherwise more supportive of palliative care.

In this qualitative interview study of PCEs, palliative care team members, and medical/surgical floor colleagues, we found that the new role of the PCE helped to “sow seeds” for successful ACP, GOC conversations, and palliative care referrals. Creating and integrating this new role was not without challenge, especially in forging new communication strategies and relationships within existing clinical workflows. Despite these challenges, the PCEs relieved existing staff from the time and emotional burdens often associated with engaging patients in ACP and GOC conversations while also approaching patients in a culturally and linguistically sensitive manner. Implementation of interventions like VIDEO-PCE require careful thought and consideration regarding integration into each hospital’s unique culture and workflows.

## Supplemental Material

Supplemental Material - The Sowers of Seeds: A Qualitative Analysis of the Role of Palliative Care Educators in Facilitating Goals-of-Care Conversations and Palliative Care Referrals Supplemental Material for The Sowers of Seeds: A Qualitative Analysis of the Role of Palliative Care Educators in Facilitating Goals-of-Care Conversations and Palliative Care Referrals by Seth N. Zupanc, BA, Lisa M. Quintiliani, PhD, Amy M. LeClair, PhD, Michael K. Paasche-Orlow, MD, Angelo Volandes, MD, MPH, Akhila Penumarthy, BS, Lori Henault, MPH, Jennifer E. Itty, MPH, Aretha D. Davis, BA, MD, JD, and Joshua R. Lakin, MD in American Journal of Hospice and Palliative Medicine®.

## Data Availability

Data from our qualitative interviews will not be shared to protect the privacy of participants.*
