# Supplementary material for: The Sowers of Seeds: A Qualitative Analysis of the Role of Palliative Care Educators in Facilitating Goals-of-Care Conversations and Palliative Care Referrals
Source: Am J Hosp Palliat Care. 2024 Aug 28;42(8):761–8. doi: 10.1177/10499091241267917 (PMC12138154; doi:10.1177/10499091241267917)
Supplement: Supplemental Material - The Sowers of Seeds: A Qualitative Analysis of the Role of Palliative Care Educators in Facilitating Goals-of-Care Conversations and Palliative Care Referrals [file sj-pdf-1-ajh-10.1177_10499091241267917.pdf]

VIDEO PCE INTERVIEW GUIDES – PCE Version

This is a semi-structured, qualitative interview guide meant to be used flexibly. The interviewer may follow-up with further open-ended questions and requests for clarification. The interviewer may also follow-up on issues raised by the participant. Questions may be asked in a different order, depending on the flow of conversation. Not all questions will be asked of each interviewee, as some questions may not be relevant to all subjects. [sub-questions are prompts]

Before beginning the interview, review consent and get the participant's verbal assent.

<u>Semi-Structured Qualitative Clinician Interviews</u>

1) First, we would like to learn some about your thoughts and experience with palliative care.
    a. How would you describe palliative care?
    b. Can you tell me about your experience with palliative care before your work as a PCE?
    c. How has your view of palliative care changed during your work as a PCE?
    d. How would you describe goals of care conversations?
    e. Can you tell me about your experience with goals of care conversations before your work as a PCE?
    f. How has your view of goals of care conversations changed during your work as a PCE?

2) Can you give us a sense of what a typical day working as a Palliative Care Educator (PCE) looks like?
    a. What are your typical activities with patients?
    b. Are you seeing patients all day, or do you have other administrative work?
    c. What has been most rewarding about the role of the PCE?
    d. What has been most challenging?
    e. Have you felt supported in your role as a PCE? If so, how?
    f. Did you feel you were a researcher or clinical staff?
    g. What was the reaction of various hospital staff to your presence?
    h. In routine practice in your setting, who is responsible for goals of care conversations?
    i. And then who typically has these conversations in reality?
    j. How are patients identified as needing to have these conversations?
    k. When you have a goal of care conversations with a patient, how do you know who know who to have these conversations with?
        i. What's your general approach to these conversations?
        ii. Has that changed over time?
    l. What happens after these conversations?
    m. How have you been using the ACP Decisions videos in your work?
    n. When did you feel a video was or was not appropriate?
    o. Which videos did you use mostly and why?
    p. If you have a challenging case, is there someone who can help you?
    q. Is there a review of cases or another way for people to get together and discuss?

3) Can you describe the processes for working with your palliative care team?

a. How do you interact with the team?
b. How frequently?
c. In what circumstances do you encourage a palliative care team consult?
d. How do you make that consult happen at your site?

4) Just a few wrap up questions:
   a. Is there anything else should I know today?
   b. Is there something that you thought I was going to ask, but didn't?

VIDEO PCE INTERVIEW GUIDES – Palliative Care and Manager Version

This is a semi-structured, qualitative interview guide meant to be used flexibly. The interviewer may follow-up with further open-ended questions and requests for clarification. The interviewer may also follow-up on issues raised by the participant. Questions may be asked in a different order, depending on the flow of conversation. Not all questions will be asked of each interviewee, as some questions may not be relevant to all subjects. [sub-questions are prompts]

Before beginning the interview, review consent and get the participant's verbal assent.

<u>Semi-Structured Qualitative Clinician Interviews</u>

1) First, we would like to learn some about your palliative care team.
    a. How many people work on the palliative care team at your site?
    b. What types of professionals do you have on your team?
    c. What kinds of palliative care services do you deliver at your site – i.e. inpatient, clinic, home-based, inpatient hospice?
    d. Are the Palliative Care Educators (PCEs) in the VIDEO PCE study a part of your palliative care team?

2) Next, we would like to understand more about how you interact with the PCEs. Could you give us a sense of your interactions with them on a typical day? Some exploratory questions:
    a. What are their typical activities with patients?
    b. Do they see patients with you or alone?
    c. How do you interact with the PCEs in a typical day?
    d. What has been most rewarding about having the PCEs as part of the team?
    e. What has been most challenging?
    f. In general, who is responsible for goals of care conversations in your setting?
    g. And then who typically has these conversations in reality?
    h. How are patients identified as needing to have these conversations?
    i. When PCEs are responsible for facilitating goals of care conversations, how are the PCE's facilitating them?
    j. How have the PCE's been facilitating interactions between the primary care teams and your palliative care team? Have you found the interactions helpful? Are you seeing patients you wouldn't have/hadn't before?
    k. Do you believe the PCE's are taking care of some tasks that may have previously led to palliative care consults? If so, how do you feel this has been going?

3) Just a few wrap up questions:
    a. Is there anything else should I know today?
    b. Is there something that you thought I was going to ask, but didn't?

VIDEO PCE INTERVIEW GUIDES – Floor Clinician Version

This is a semi-structured, qualitative interview guide meant to be used flexibly. The interviewer may follow-up with further open-ended questions and requests for clarification. The interviewer may also follow-up on issues raised by the participant. Questions may be asked in a different order, depending on the flow of conversation. Not all questions will be asked of each interviewee, as some questions may not be relevant to all subjects. [sub-questions are prompts]

Before beginning the interview, review consent and get the participant's verbal assent.

<u>Semi-Structured Qualitative Clinician Interviews</u>

1) First, we would like to learn some about the floor you work on.
   a. Can you describe the location in the hospital where you work?
   b. What kinds of patients do you typically see?

2) Next, we would like to learn some about your thoughts and experience with palliative care.
   a. How would you define palliative care?
   b. How often are you engaging palliative care in the care of your patients?
   c. What barriers exist to engaging palliative care for your patients?
   d. Now, how do you define goals of care conversations?
   c. How often are you having goals of care conversations with your patients?
   d. What barriers exist to having goals of care conversations with your patients?

4) Next, we would like to understand more about how you interact with the PCEs. Could you give us a sense of your interactions with them on a typical day? Some exploratory questions:
   a. Can you describe the role of the PCE as you understand it?
   b. Can you share some examples of interactions you have had with them?
   c. What are their typical activities with patients?
   d. Do they see patients with you or alone?
   e. How do you interact with the PCEs in a typical day?
   f. What has been most rewarding about having the PCEs as part of the team?
   g. What has been most challenging?
   h. In general, who is responsible for goals of care conversations in your setting?
   i. And then who typically has these conversations in reality?
   j. How are patients identified as needing to have these conversations?
   k. How are the PCE's facilitating them?
   l. How have the PCE's been facilitating interactions between your team and the palliative care team? Have you found the interactions helpful?

3) Just a few wrap up questions:
   a. Is there anything else should I know today?
   b. Is there something that you thought I was going to ask, but didn't?
